# Appropriate reference region selection of ^18^F-florbetaben and ^18^F-flutemetamol beta-amyloid PET expressed in Centiloid

**DOI:** 10.1038/s41598-020-70978-z

**Published:** 2020-09-11

**Authors:** Soo Hyun Cho, Yeong Sim Choe, Seongbeom Park, Young Ju Kim, Hee Jin Kim, Hyemin Jang, Seung Joo Kim, Jun Pyo Kim, Young Hee Jung, Byeong C. Kim, Duk L. Na, Seung Hwan Moon, Sang Won Seo

**Affiliations:** 1 Department of Neurology, Samsung Medical Center, Sungkyunkwan University School of Medicine, 81 Irwon-ro, Gangnam-gu, Seoul, 06351 Republic of Korea; 2 Department of Neurology, Chonnam National University Hospital, Chonnam National University Medical School, Gwangju, Republic of Korea; 3 Department of Health Sciences and Technology, SAIHST, Sungkyunkwan University, Seoul, Republic of Korea; 4 Neuroscience Center, Samsung Medical Center, Seoul, Republic of Korea; 5 Department of Neurology, Gyeongsang National University School of Medicine, Gyeongsang National University Changwon Hospital, Changwon, Republic of Korea; 6 Department of Neurology, Myoungji Hospital, Hanyang University, Goyangsi, Republic of Korea; 7 Stem Cell and Regenerative Medicine Institute, Samsung Medical Center, Seoul, Republic of Korea; 8 Department of Nuclear Medicine, Samsung Medical Center, Sungkyunkwan University School of Medicine, 81 Irwon-ro, Gangnam-gu, Seoul, 06351 Republic of Korea; 9 Department of Clinical Research Design and Evaluation, SAIHST, Sungkyunkwan University, Seoul, Republic of Korea; 10 Samsung Alzheimer Research Center, Samsung Medical Center, Seoul, Republic of Korea; 11 Center for Clinical Epidemiology, Samsung Medical Center, Seoul, Republic of Korea

**Keywords:** Biomarkers, Medical research, Neurology

## Abstract

The Centiloid (CL) is a method for standardizing amyloid beta (Aβ) quantification through different ligands and methods. To find the most appropriate reference region to reduce the variance in the Aβ CL unit between ^18^F-florbetaben (FBB) and ^18^F-flutemetamol (FMM), we conducted head-to-head comparisons from 56 participants using the direct comparison of FBB-FMM CL (dcCL) method with four reference regions: cerebellar gray (CG), whole cerebellum (WC), WC with brainstem (WC + B), and pons. The FBB and FMM dcCL units were highly correlated in four reference regions: WC (R^2^ = 0.97), WC + B (R^2^ = 0.98), CG (R^2^ = 0.92), and pons (R^2^ = 0.98). WC showed the largest effect size in both FBB and FMM. Comparison of the variance of the dcCL values within the young control group showed that with FBB, WC + B had the smallest variance and with FMM, the WC had the smallest variance. Additionally, WC + B showed the smallest absolute difference between FBB and FMM, followed by the WC, pons, and CG. We found that it would be reasonable to use the WC or WC + B as the reference region when converting FBB and FMM SUVRs into dcCL, which can increase the accuracy of standardizing FBB and FMM PET results.

## Introduction

Amyloid β (Aβ) PET imaging using ^11^C-Pittsburgh Compound-B (PiB)^1^, ^18^F-florbetapir^2^, ^18^F-florbetaben (FBB)^3^, and ^18^F-flutemetamol (FMM)^4^ has been developed to measure the accumulation of fibrillar beta-Aβ deposition in vivo. The most commonly used quantification method in brain Aβ PET imaging is the standardized uptake value (SUV) ratio (SUVR), a relative measurement defined as the ratio of SUV in the target region to the SUV in a reference region.

The identification of an appropriate reference region is of great importance in clinical trials that need accurate measurements of Aβ accumulation as a biomarker for Alzheimer’s disease (AD). A suitable reference region has to be free of Aβ and have the same non-displaceable activity as the target area^5^. If the reference region SUV is high due to accumulated Aβ or spillover of the signal from adjacent tissue, the SUVR of the target region will be low, which falsely reduces the measure of Aβ burden. Previous studies have used the cerebellar gray matter (CG), whole cerebellum (WC), whole cerebellum and brainstem (WC + B), and pons as reference regions.

The appropriate reference region can differ with the type of ligand because the ^18^F-ligands differ in their stability over time^1^ and comparable blood flow^6^. For this reason, various studies have used different reference regions with different kinds of ligands^7–9^. Although surveys have been done to investigate which reference region is most appropriate for particular ligands when calculating the SUVR, no research has been conducted to determine which reference is appropriate for the Centiloid measurement of F-labeled ligands. The Centiloid (CL) method enables not only standardized PET-based measurement of the Aβ burden but also comparisons of the effect size between AD patients and young controls (YCs) and the variability of Aβ found in the YC group with different types of ligand^10^. The variability between Aβ in YCs, who can be expected to be pathology-free, and Aβ in patients provides an estimate of the relative precision of two tracers used to derive CL. Furthermore, an appropriate reference region could be expected to show the smallest CL difference between ligands in a single subject.

In this study, we investigated which reference regions are most appropriate to calculate CL unit when using the FBB and FMM ligands. We used head-to-head comparisons of Aβ PET with the FBB and FMM ligands using a direct comparison of the FBB-FMM CL (dcCL) method^11^ with four reference regions: CG, WC, WC + B, and pons, which have been frequently used in previous studies. We hypothesized that we would find differences in the appropriateness of the different reference regions between FBB and FMM in terms of the largest effect size between the AD and YC groups and the smallest variability within the YC group. We further hypothesized that the absolute dcCL differences between FBB and FMM might vary among the reference regions in single subjects.

## Results

### Participant demographics

Demographic characteristics of the participants are described in Table 1. The old controls (OCs) were older than the Alzheimer's disease-related cognitive impairment (ADCI) participants. There was no difference in sex between the three diagnostic groups. The Mini-Mental State Examination (MMSE) scores of the ADCI participants differed significantly from those of both the OCs and YCs, but the OCs and YCs did not differ from each other. The freqency of *APOE* ε4 carriers was 80% among the Aβ(+) ADCI participants, 18.8% among the Aβ(−) OCs, and 15% among the Aβ(−) YCs.

**Table 1:** Participant demographics and clinical findings.

	Aβ(+) ADCI	Aβ(−) OC	Aβ(−) YC
Number of participants	20	16	20
Sex (M/F)	9/11	6/10	11/9
Age, years (mean ± SD)	67.2 ± 8.2	74.0 ± 3.6^a,b^	32.0 ± 3.9^a,b^
MMSE (mean ± SD)	19.2 ± 7.0	26.9 ± 2.1^a^	29.9 ± 0.4^a^
*APOE* ε4, no. (%)	16 (80)	3 (18.8)^a^	3 (15)^a^

Statistical analyses used chi-square tests for sex and *APOE* ε4. Analysis of variance was used for age and MMSE.

*Aβ* amyloid beta, *ADCI* Alzheimer’s disease-related cognitive impairment, *OC* old control, *YC* young control, *MMSE* Mini-Mental State Examination, APOE ε4 apolipoprotein E ε4 allele, *SD* standard deviation, *M/F* male/female.

^a^*p* < 0.05 between Aβ(+) ADCI and Aβ(−) OC or Aβ(−) YC.

^b^*p* < 0.05 between Aβ(−) OC and Aβ(−) YC.

### Correlation of SUVRs between FBB and FMM in the global cortical target (CTX) volume of interest (VOI)

**Figure 1:**
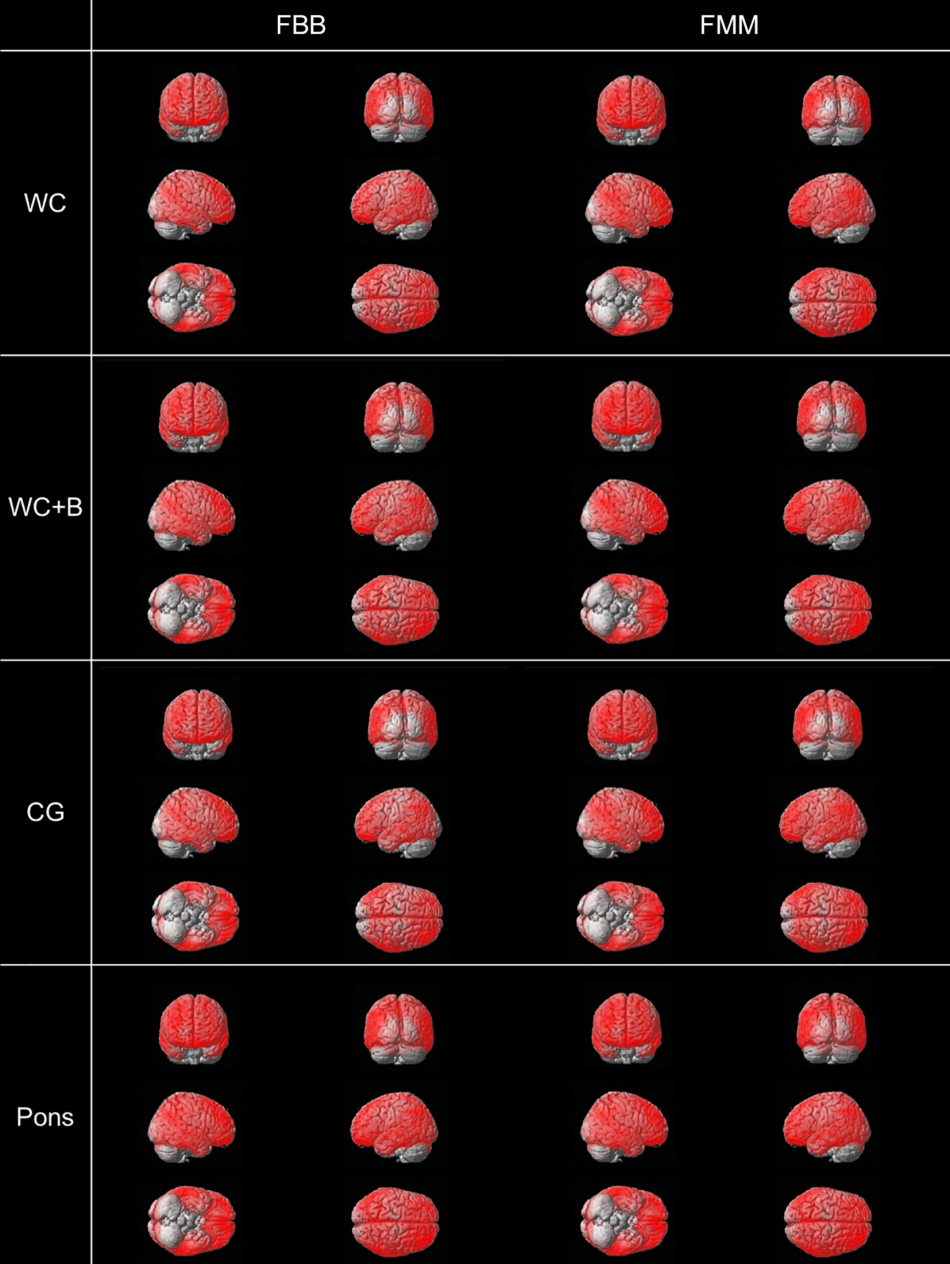
Comparison of the amyloid β (Aβ) binding patterns between the ADCI and OC groups (false discovery rate [FDR] corrected *p* < 0.05). ADCI participants has significantly higher Aβ binding than the OCs in each reference region. There is no significant difference in FBB and FMM between the ADCI and OC Aβ deposition patterns. Abbreviations: *FBB* ^18^F-florbetaben, *FMM* ^18^F-flutemetamol, *WC* whole cerebellum, *WC* + *B* whole cerebellum + brainstem, *CG* cerebellar gray matter, *ADCI* Alzheimer’s disease-related cognitive impairment, *OC* old control.

The ADCI participants had significantly increased SUVRs for FBB and FMM in the frontal, parietal, temporal, and precuneus area compared with the OCs, which was similar across the four reference regions (false discovery rate [FDR] corrected *p* < 0.05, Fig. 1).

**Figure 2:**
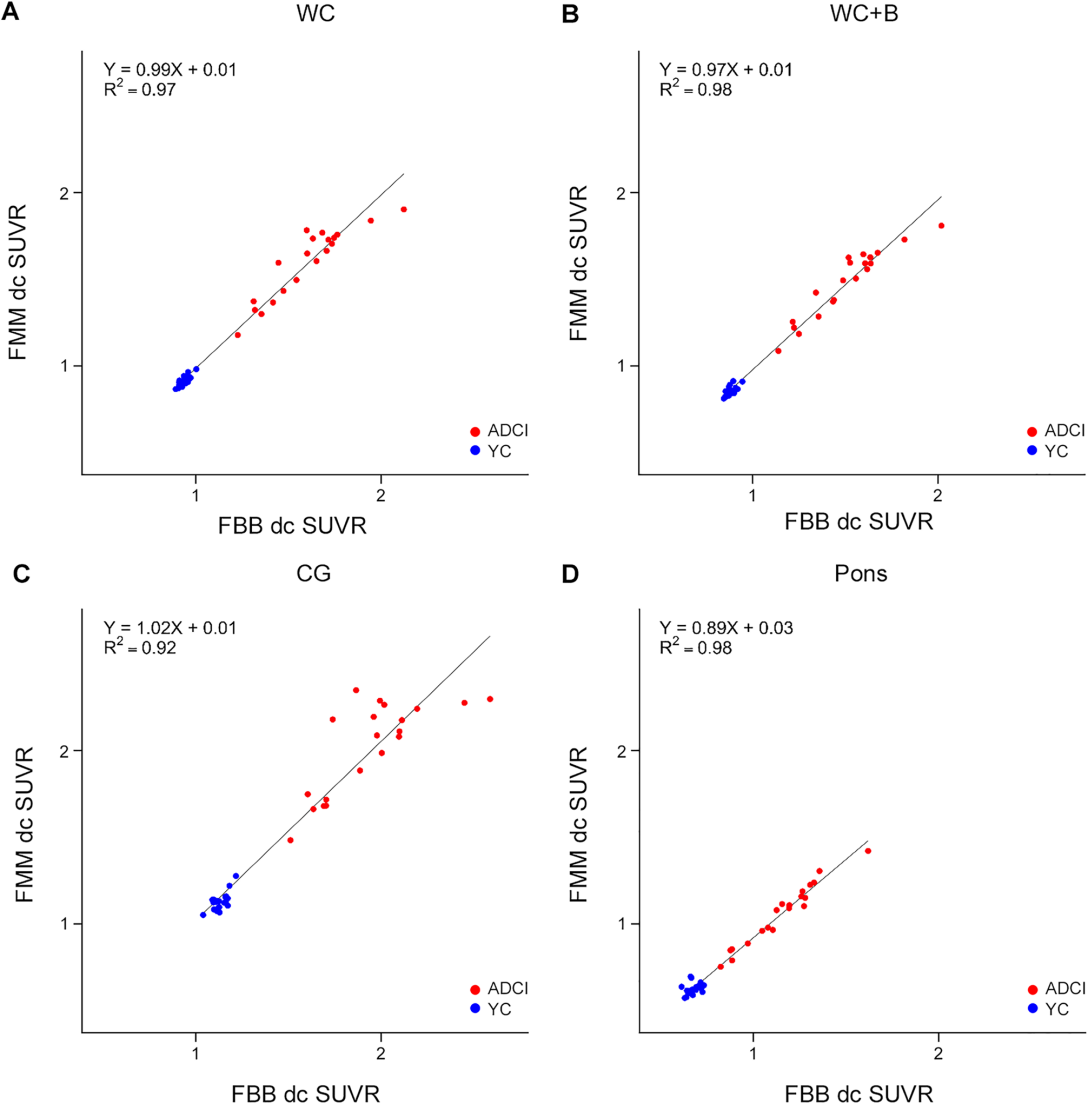
Plot of the paired ^18^F-FMM SUVR and ^18^F-FBB SUVR with the FBB-FMM global cortical target (CTX) volume of interest (VOI) for each reference region. SUVR correlation for each subject calculated by the standard Centiloid method with a standard large single CTX VOI with reference to the (**a**) whole cerebellum (WC), (**b**) whole cerebellum + brain stem (WC + B), (**c**) cerebellar gray matter (CG) and (**d**) pons. Regression equations are shown for each scatterplot. Abbreviations: *ADCI* Alzheimer’s disease-related cognitive impairment, *YC* young control, *FBB* ^18^F-florbetaben, *FMM* ^18^F-flutemetamol, *SUVR* standardized uptake value ratio, *CTX VOI* global cortical target volume of interest.

The paired FBB and FMM images were then analyzed against the FBB-FMM CTX-VOI template and demonstrated excellent linear correlation (Fig. 2). The SUVRs for FBB and FMM in the CTX VOI correlated highly in all four reference regions: WC (R^2^ = 0.97), WC + B (R^2^ = 0.98), CG (R^2^ = 0.92), and pons (R^2^ = 0.98). The range of slopes for the FBB–FMM SUVR associations was also similar across all reference regions: WC (0.99), WC + B (0.97), CG (1.02), and pons (0.89).

### Correlation of dcCL units between FBB and FMM

**Figure 3:**
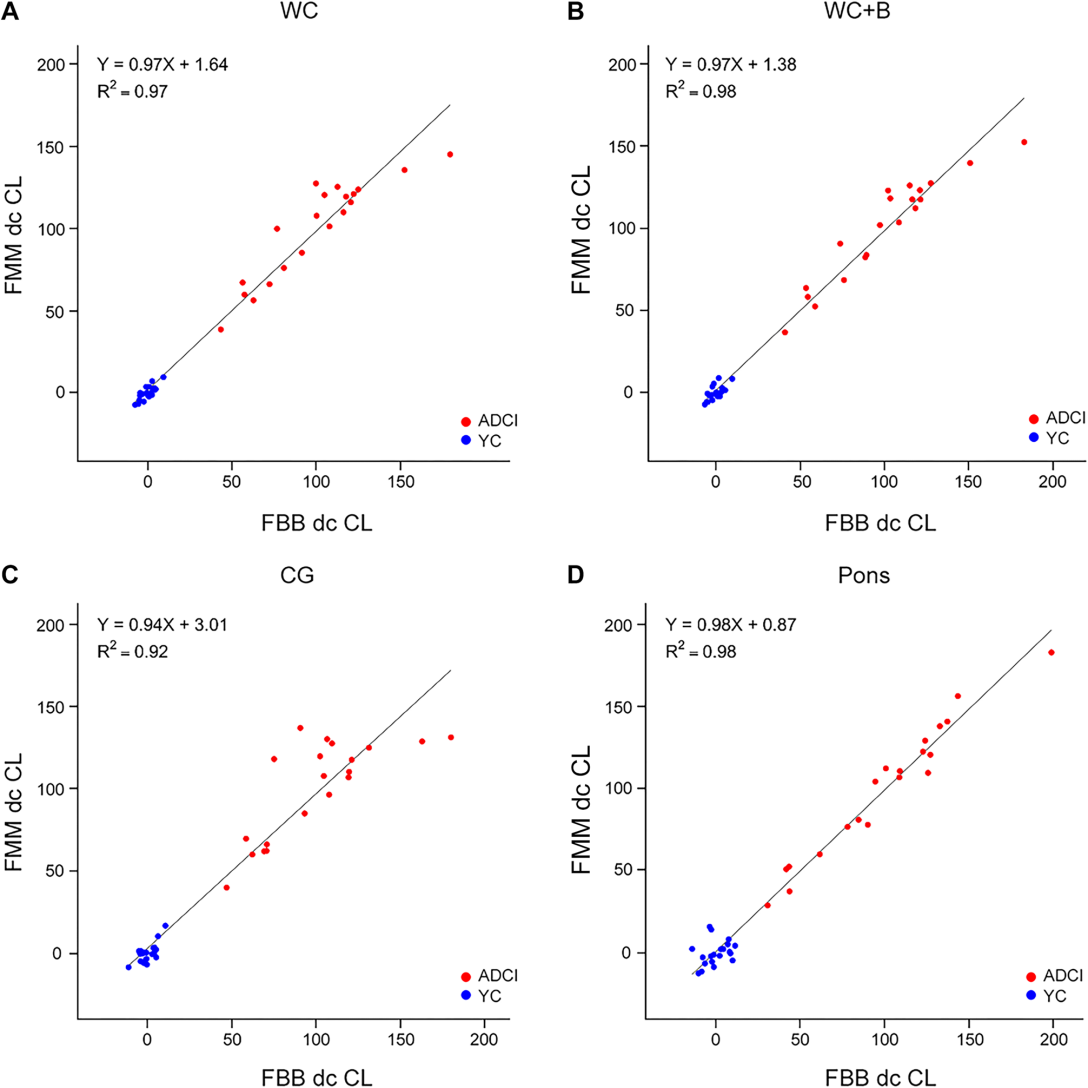
Plot of the direct comparison of FBB-FMM CL (dcCL) units between ^18^F-FBB and ^18^F-FMM for each reference region. The dcCL unit correlation for each subject calculated for the FBB-FMM global cortical target region volume of interest (CTX VOI) with reference to the (**a**) whole cerebellum (WC), (**b**) whole cerebellum + brain stem (WC + B), (**c**) cerebellar gray matter (CG), and (**d**) pons. Regression equations are shown for each scatterplot. Abbreviations: *ADCI* Alzheimer’s disease-related cognitive impairment, *YC* young control; *FBB* ^18^F-florbetaben, *FMM* ^18^F-flutemetamol, *SUVR* standardized uptake value ratio, *dcCL* the Centiloid (CL) units using a direct comparison of the FBB-FMM CL method.

Both the SUVR_FBB_ and SUVR _FMM_ values from the FBB-FMM CTX VOI were converted directly into dcCL units. A direct relationship was thus formed between the FBB or FMM SUVRs and the dcCL units in the four different reference regions, as summarized in Table 2. The dcCL units between FBB and FMM also correlated highly in all four reference regions (Fig. 3), WC (R^2^ = 0.97), WC + B (R^2^ = 0.98), CG (R^2^ = 0.92), and pons (R^2^ = 0.98), and the range of slopes for the FBB–FMM dcCL associations was similar across all reference regions: WC (0.97), WC + B (0.97), CG (0.94), and pons (0.98).

**Table 2:** Equations for Centiloid depend on the reference region with FBB and FMM.

Reference region	FBB	FMM
WC	dcCL_FBB_ = 151.4 × SUVR_FBB_ − 142.2	dcCL_FMM_ = 148.5 × SUVR_FMM_ − 137.1
WC + B	dcCL_FBB_ = 160.9 × SUVR_FBB_ − 143.0	dcCL_FMM_ = 161.7 × SUVR_FMM_ − 139.7
CG	dcCL_FBB_ = 123.1 × SUVR_FBB_ − 139.2	dcCL_FMM_ = 113.5 × SUVR_FMM_ − 128.8
Pons	dcCL_FBB_ = 211.1 × SUVR_FBB_ − 144.8	dcCL_FMM_ = 232.8 × SUVR_FMM_ − 148.5

*FBB* ^18^F-florbetaben, *FMM* ^18^F-flutemetamol, *SUVR* standardized uptake value ratio, *WC* whole cerebellum, *CG* cerebellar gray matter, *WC* + *B* whole cerebellum + brainstem, *dcCL* Centiloid unit from direct comparison of the FBB-FMM CL method.

**Table 3:** Summary statistics of SUVR and Centiloid scores in disease-specific cortical target regions with each of four reference regions.

Reference region	SUVR				Centiloid units (dcCL)			
	WC	WC + B	CG	Pons	WC	WC + B	CG	Pons
**FBB**								
*ADCI*								
Mean	1.5998	1.5104	1.9431	1.1598	100.00	100.00	100.00	100.00
SD	0.2221	0.2162	0.2775	0.1997	33.63	34.78	34.16	42.15
*YC*								
Mean	0.9394	0.8888	1.1306	0.6860	0.00	0.00	0.00	0.00
SD	0.0280	0.0260	0.0408	0.0349	4.23	4.18	5.02	7.36
Effect size	4.07	3.94	3.99	3.22	4.07	3.94	3.99	3.22
**FMM**								
*ADCI*								
Mean	1.5964	1.4821	2.0164	1.0678	100.00	100.00	100.00	100.00
SD	0.2028	0.1951	0.2646	0.1761	30.12	31.56	30.02	41.00
*YC*								
Mean	0.9230	0.8638	1.1350	0.6381	0.00	0.00	0.00	0.00
SD	0.0290	0.0271	0.0508	0.0319	4.31	4.39	5.76	7.43
Effect size	4.53	4.33	4.51	3.31	4.53	4.33	4.51	3.31

*FBB* ^18^F-florbetaben, *FMM* ^18^F-flutemetamol, *ADCI* Alzheimer’s disease-related cognitive impairment, *YC* young control, *SUVR* standardized uptake value ratio, *WC* whole cerebellum; *WC* + *B* whole cerebellum + brainstem, *CG* cerebellar gray matter, *dcCL* Centiloid unit from direct comparison of the FBB-FMM CL method.

### Effect size of dcCL values between the ADCI and YC groups in four reference regions

The differences in the dcCL values between the ADCI and YC groups are represented as effect sizes (Table 3). Using the FBB ligand, the highest effect size between the ADCI and YC groups was generated using the WC (4.07) as the reference region, followed by the CG (3.99), WC + B (3.94), and pons (3.22). Using the FMM ligand, the highest effect size between the ADCI and YC groups was generated using the WC (4.53), followed by the CG (4.51), WC + B (4.33), and pons (3.31).

### Variance of dcCL values within the YC group

The variance in the dcCL values is shown in Table 3. For FBB, the smallest variances in the dcCL values of the YCs were found when using WC + B (4.18) as the reference region, followed in order by the WC (4.23), CG (5.02), and pons (7.36). For FMM, the smallest variances in the dcCL values of the YCs were observed when using the WC (4.31) as the reference region, followed by WC + B (4.39), CG (5.76), and pons (7.43). Leven’s test for equal variance indicated differences in the variance of dcCL values among the four reference regions with the FBB ligand (*p* = 0.01). Compared with WC + B, only pons showed significantly greater variance in the dcCL values of the YCs (*p* = 0.02) with the FBB ligand. No differences occurred in the variances of the dcCL values in the YCs (*p* = 0.17) with the FMM ligand.

### Absolute differences in dcCL units between FBB and FMM

**Figure 4:**
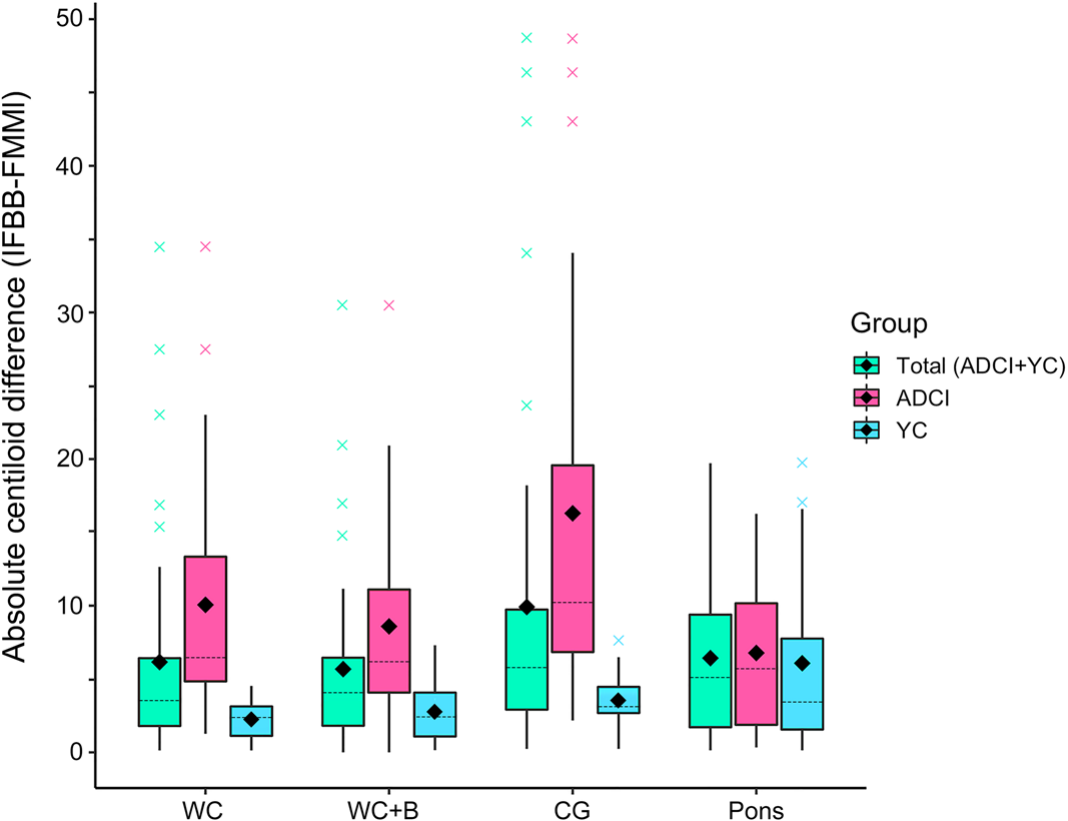
Box plots of the absolute Centiloid unit difference between FBB and FMM obtained with each reference and group. This box plot represents the absolute dcCL unit differences between FBB and FMM with four different reference regions in the total, ADCI and YC groups. The diamond in the box plot means mean and the dotted line means median. Abbreviations: *FBB* ^18^F-florbetaben, *FMM* ^18^F-flutemetamol, *dcCL* the Centiloid (CL) units using the direct comparison of FBB-FMM CL method, *WC* whole cerebellum, *WC* + *B* whole cerebellum + brainstem, *CG* cerebellar gray matter.

The distribution of absolute dcCL differences between the two ligands within the groups and by reference region are presented as box plots (Fig. 4). We compare the absolute value differences in the dcCL units between FBB and FMM by using the generalized estimating equation (GEE) in total group. The smallest mean absolute differences by reference region in total group (ADCI + YC) were WC + B (5.68 [0.84], mean [standard error]), followed in order by the WC (6.16 [1.01]), pons (6.43 [0.87]), and CG (9.91 [1.64]). There was a difference among the references (*p* < 0.001). Specifically, the WC + B and CG showed significant absolute dcCL unit differences between the two ligands (*p* < 0.001). When comparing the absolute dcCL difference between the ADCI and YC groups, the ADCI group (10.43 [1.76]) demonstrated a significant larger difference than the YC group (3.65 [0.52]) (*p* < 0.001).

## Discussion

To determine which reference region might be appropriate when we combine the FBB and FMM ligands for Aβ imaging, we assessed dcCL units using four different reference regions by making head-to-head comparisons. Our major findings are as follows. First, both FBB and FMM had the largest effect size for the dcCL unit when the WC was used as the reference region, followed in order by the CG, WC + B, and pons. Second, regarding the variance in dcCL values within the YC group, WC + B was the reference region with the smallest variance for FBB, followed in order by the WC, CG, and pons. Only pons had significantly higher dcCL variations of within the YC group compared with WC + B. For FMM, WC had the smallest variance followed in order by the WC + B, CG, and pons. Third, the smallest absolute differences between the dcCL units for the two ligands and the reference regions were with the WC + B, followed in order by the WC, pons, and CG. Compared with the WC + B, the CG showed significantly larger absolute dcCL unit differences between the two ligands. Taken together, our findings suggest that it would be reasonable to use the WC or WC + B as the reference region when converting FBB and FMM SUVRs into dcCL. Through this study, we are able to propose an appropriate reference region for obtaining dcCL, which can increase the accuracy of standardizing FBB and FMM PET results.

Our first major finding is that both FBB and FMM had the largest effect size in terms of dcCL units when the WC was used as the reference region, followed by the CG, WC + B and pons. That finding is consistent with a previous study^10^ that transformed PiB SUVRs into CL units. That is, the WC showed the largest effect size for PiB ligands, and the WC + B was nearly as good. On the other hand, the pons showed the smallest effect size^10^. The reason why the largest effect size was with the WC and the smallest was with the pons, regardless of the type of amyloid ligand used, is important but uncertain. Because the WC includes white matter, it could have higher signal intensity and less susceptibility to noise, and it could include tissue less vulnerable to edge and truncation effects. On the other hand, the pons might introduce variability because of its small volume and sensitivity to head motion and the poor performance of many normalization routines^10,12^. Unlike the WC, the pons might be susceptible to scatter and truncation effects because it is located at the outer extremes of the field of view of an axial PET scanner^9^.

Our second major finding is that in the variances of the dcCL values within the YC group, which provide an estimate of the relative precision of the two tracers, the WC + B (FBB) and WC (FMM) showed the smallest variance, and both FBB and FMM showed the largest variance in the pons. With FBB in particular, the pons had the largest dcCL variation within the YC group by a significant margin compared with the WC + B which showed the smallest dcCL variation within the YC group. This finding is consistent with a previous study that transformed PiB SUVR into CL units, which found that the pons had the largest variance in YC CL, and the WC had the smallest variance in YC CL. Therefore, our findings suggest that the pons should be used carefully as a reference when converting FBB SUVR into CL units.

Our third major finding is that the reference region with the smallest absolute dcCL differences between FBB and FMM within a single subject was the WC + B, and the region with the largest difference was the CG. The reason why the differences in dcCL units between FBB and FMM varied with the reference region is important but unclear. Especially, the differences are driven from the ADCI group rather than control participants. Although the reference regions were classified as Thal 5, Aβ also accumulates in those regions in patients with advanced disease. Aβ burdens that accumulate in these reference regions include diffuse plaques, neuritic plaques, and cerebral amyloid angiopathy^13^. Both cortical neuritic (Bielschowsky) and total (Aβ immunohistochemistry) plaque burden was higher with advancing disease progression, as determined by the Aβ phase^14,15^. The probability that a PET image would be rated as abnormal increased with neocortical neuritic plaque frequency and an AD diagnosis^14,15^. Although the primary form of Aβ pathology detected with FBB and FMM PET imaging is neuritic plaque burden^16,17^, the PET signal is also affected by the presence of diffuse plaques^16^. The degree of neuritic/diffuse plaque accumulation in the reference regions and the affinity of the tracers for those plaques could be major factors influencing the conversion to CL units between FBB and FMM. The potential influence of cerebellar Aβ burden on cortical CL units when the CG is the reference region needs to be investigated further^18^.

Our study has the advantage of direct comparisons through head-to-head studies with FBB and FMM ligands and four different reference regions. However, our study also has limitations. First, our results might be difficult to apply to all Aß PET scans because of differences among Aß PET systems and reconstruction methods at other sites. Second, we were unable to evaluate the correlation between postmortem pathology and CL units, so our results are not based on neuropathological studies. More precise studies of reference regions based on pathology should be performed. Third, due to the small number of head-to-head participants, our study should be repeated with more participants to replicate our results. Despite those limitations, the results of this study will be helpful in standardizing a CL method for FBB-FMM PET.

In conclusion, we performed a head-to-head comparison for converting FBB and FMM PET SUVRs into CL units using different reference regions. We found that it would be reasonable to use the WC or WC + B as the reference region when converting FBB and FMM SUVRs into dcCL, but careful attention must be paid to the ligand and reference region.

## Methods

### Participants

We recruited 20 YCs (younger than 40 years), 16 OCs (older than 65 years), and 20 Aβ PET positive (+) patients, 16 with AD dementia and 4 with amnestic mild cognitive impairment (aMCI), which we denoted as the ADCI group^19^. All participants underwent Aβ PET with both FBB and FMM, as well as magnetic resonance imaging (MRI). AD demenita participants were diagnosed based on the National Institute on Aging-Alzheimer’s Association criteria for probable AD^20^. To be diagnosed with aMCI, participants had to meet Petersen’s criteria^21^ and show objective memory impairment one standard deviation below the norm in at least one memory test^22^. The OCs were all characterized by the following: (1) no history of neurologic or psychiatric disorders, (2) normal cognitive function determined using neuropsychological tests, and (3) Aβ negative on PET image. Healthy YCs had (1) no history of neurologic or psychiatric disorders, (2) normal cognitive function (MMSE score), and (3) Aβ negative on PET image.

All participants underwent clinical interview and neurological and neuropsychological examinations and laboratory testing: complete blood count, blood chemistry, syphilis serology, thyroid function tests and vitamin B12/folate. Brain MRI confirmed the absence of structural lesions to rule out other causes of cognitive impairment including territorial cerebral infarctions, brain tumors and hippocampal sclerosis.

This study protocol was approved by the Institutional Review Board of Samsung Medical Center and carried out in accordance with the approved guidelines. We obtained written informed consent obtained from participants’ parents/legally authorized representatives.

### MRI data acquisition

All participants underwent three-dimensional, T1, turbo, field-echo images on the same 3.0 T MRI scanner (Philips Achieva; Philips Healthcare, Andover, MA, USA) using the following imaging parameters: sagittal slice thickness, 1.0 mm with 50% overlap; no gap; repetition time of 9.9 ms; echo time of 4.6 ms; flip angle of 8°; and matrix size of 240 × 240 pixels reconstructed to 480 × 480 over a field of view of 240 mm.

### Aβ PET data acquisition

Participants underwent FBB PET and FMM PET at Samsung Medical Center using a Discovery STe PET/CT scanner (GE Medical Systems, Milwaukee, WI, USA) in three-dimensional scanning mode. We examined 47 slices of 3.3-mm thickness spanning the entire brain. The paired FBB and FMM PET images were acquired on two separate days; there were no differences in the mean interval times (4.0 ± 2.5 months across all groups) among the three groups (*P* = 0.89). We performed FBB PET first in half of the participants (total 29; 8 ADCI, 12 OCs, and 9 YCs) and FMM PET first in the other half (total 27; 12 ADCI, 4 OCs, and 11 YCs). CT images were acquired using a 16-slice helical CT (140 keV, 80 mA; 3.75-mm section width) for attenuation correction. According to the protocols proposed by the ligands’ manufacturers, a 20-min emission PET scan in dynamic mode (4 × 5 min frames) was performed 90 min after the injection of a mean dose of 311.5 MBq of FBB or 185 MBq of FMM. Three-dimensional (3D) PET images were reconstructed in a 128 × 128 × 48 matrix with a voxel size of 2 mm × 2 mm × 3.27 mm using the ordered-subsets expectation maximization algorithm (FBB iterations = 4 and subsets = 20; FMM iterations = 4 and subsets = 20). All PET images were reviewed by nuclear medicine physicians blinded to patient information who diagnosed the classification and dichotomized the images as Aβ positive or negative using visual reads^23,24^.

### Aβ PET imaging analysis

We replicated the image processing steps described in the previously published Centiloid Project^10^. Each participant’s MRI was co-registered to the MNI-152 template, and then each participant’s PET image was co-registered via the derived MRI transformation parameters using the SPM8 unified segmentation method, as described in detail in the CL methodology paper^10^. We used T1-weighted MR-image correction with the N3 algorithm only for intensity non-uniformities^25^. No corrections were applied to the PET images for brain atrophy or partial volume effects. We downloaded the WC, WC + B, CG, and pons masks from the Global Alzheimer’s Association Interactive Network (GAAIN) website (https://www.gaain.org).

### FBB-FMM CTX VOI

The FBB-FMM CTX VOI was generated using SUVR parametric images (and the WC reference VOI) from the 20 typical ADCI patients (AD-CTX), who were also used in the CL scaling described below, as well as the 16 OCs (OC-CTX). We applied the same method as in the published CL Project paper^10^. To generate the FBB-FMM CTX VOI, the average OC-CTX image was subtracted from the average AD-CTX image. Then, we defined the FBB-FMM CTX VOI as areas of AD-related brain Aβ accumulation common to both FBB PET and FMM PET. To extract the FBB-FMM CTX VOI, we first obtained the FBB CTX VOI and FMM CTX VOI and then found the intersection of those images. After that, only the upper 20% of the intersection images were used as the FBB-FMM CTX VOI^26^. We smoothed those regions with a 3D-Gaussian filter with a full width at half maximum of 8.0 mm to retain regions similar to the published CTX region-of-interest^10^. The individual SUVR values for the FBB-FMM CTX VOI were calculated using the WC as the reference region.

### Centiloid units

As part of our dcCL method for FBB and FMM PET without conversion to PiB^11^, we converted the SUVR values of the FBB-FMM CTX VOI directly into dcCL units using the following CL conversion equation^10^:

$$CL = 100 \times \left( {SUVR_{ind} - SUVR_{YC - 0} } \right) / \left( {SUVR_{ADCI - 100} - SUVR_{YC - 0} } \right)$$

where SUVR_ind_ represents the individual SUVR values of all YC-0 and ADCI-100 participants, and SUVR_YC-0_ and SUVR_ADCI-100_ represent each group’s mean SUVR values. Each CL equation was derived uniquely for FBB and FMM PET and published recently^11^. Therefore, we applied those equations separately to the FBB (dcCL_FBB_ = 151.42 × SUVR_FBB_ − 142.24) and FMM (dcCL_FMM_ = 148.52 × SUVR_FMM_ − 137.09) SUVR from the FBB-FMM CTX VOI.

### Statistical analyses

To compare the demographic characteristics, we used analysis of variance for continuous variables and chi-square testing for categorical variables. To compare the Aβ binding pattern between the ADCI participants and OCs in each reference region and with each ligand, we used analysis of covariance (covariate: age) in each reference region with each tracer and a voxel-based statistical analysis in SPM 8 and Matlab 2014b. We used the FDR correction with a significance level of 0.05. We also performed a linear regression to assess correlations between the cortical retention values between FBB and FMM.

To find the most appropriate reference region, we proceeded through the following steps with three methods. First, selection of the standard reference region was based on the effect size of the difference between the AD-100 and YC-0 groups in the variance of the reference regions. The effect size was calculated using the following equation:

$${\text{Effect Size}} = \left( {\mu_{p} - \mu_{n} } \right)/\sqrt {(N_{p} \sigma_{p}^{2} + N_{n} \sigma_{n}^{2} )/\left( {N_{p} + N_{n} - 2} \right)}$$

where μ_p_ and μ_n_ are the average SUVR in the ADCI and YC groups; σ_p_^2^ and σ_n_^2^ are the variance of the ADCI and YC groups; N_p_ and N_n_ are the number of participants in the ADCI and YC groups.

Second, we did Leven’s test for equal variance and the post-hoc test to find the within-group variability for each ligand. Third, we compared the absolute value differences in the dcCL units between FBB and FMM. For this analysis, we used the generalized estimating equation (GEE). We used SPSS version 22.0 (SPSS Inc., Chicago, IL, USA) for the chi-square testing, Spearman correlation, Leven’s test, and GEE.

## Data Availability

The data that support the findings of this study are available from the corresponding author upon reasonable request.
